# Supplementary material for: Gender- and Grade-Dependent Activation of Androgen Receptor Signaling in Adult-Type Diffuse Gliomas: Epigenetic Insights from a Retrospective Cohort Study
Source: Biomedicines. 2025 Sep 28;13(10):2379. doi: 10.3390/biomedicines13102379 (PMC12561669; doi:10.3390/biomedicines13102379)
Supplement: Supplementary file 1 [file biomedicines-13-02379-s001.zip › biomedicines-3813143-supplementary.pdf]

Suppl. Figure S1A) Comparative analysis of DNA methylation in gliomas with high vs. low immunohistochemical expression of AR: *AR* CAG REPEATS (promoter)

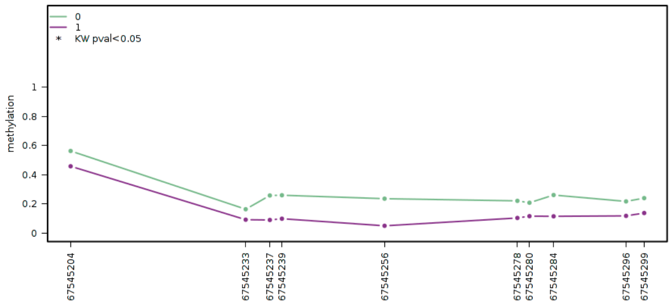

AR CAG repeats Males

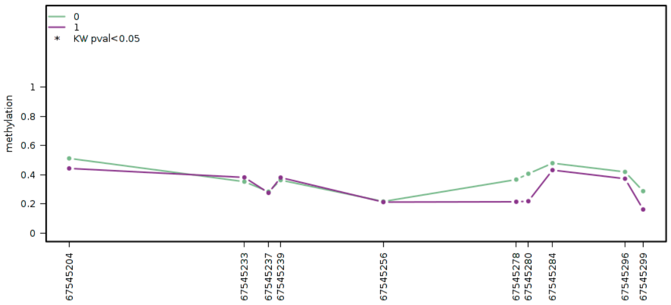

AR CAG repeats Females

Copyright: © 2025 by the authors. Licensee MDPI, Basel, Switzerland. This article is an open access article distributed under the terms and conditions of the Creative Commons Attribution (CC BY) license (https://creativecommons.org/licenses/by/4.0/).

Suppl. Figure S1B) Comparative analysis of DNA methylation in gliomas with high vs. low immunohistochemical expression of AR: *AR* INTRA CAG-CGG REPEATS (promoter).

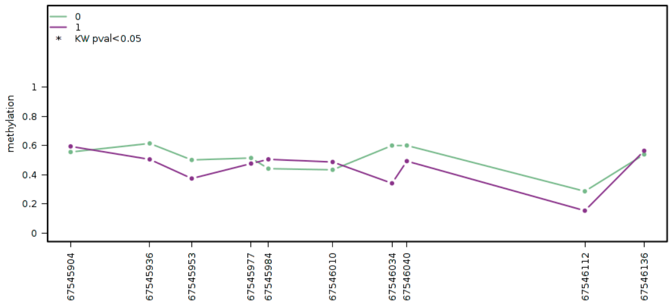

AR INTRA CAG-CGG repeats Males

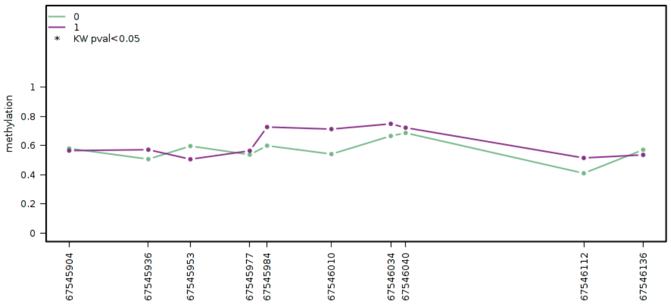

AR INTRA CAG-CGG repeats Females

Suppl. Figure S1C) Comparative analysis of DNA methylation in gliomas with high vs. low immunohistochemical expression of AR: *AR* BEFORE CGG REPEATS (promoter)

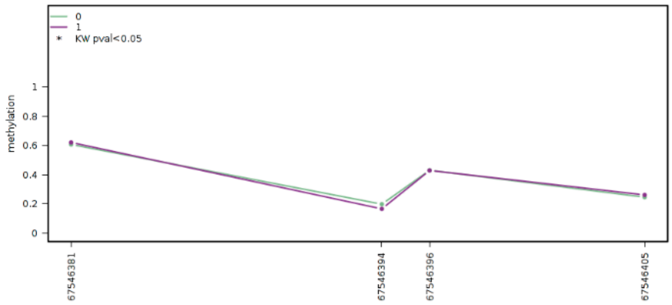

AR BEFORE CGG REPEATS repeats Males

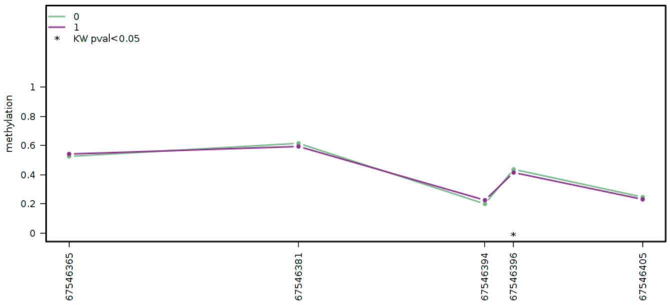

AR BEFORE CGG REPEATS repeats Females

 https://doi.org/10.3390/biomedicines13102379

Suppl. Figure S1D) DNA methylation in gliomas with high vs. low immunohistochemical expression of AR: *AR* CGG REPEATS (promoter).

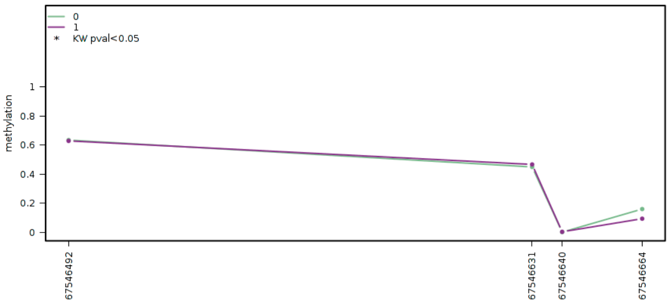

AR CGG repeats Males

Suppl. Figure S1 A,B,C,D. DNA methylation for different *AR* gene promoter regions compared between two groups (positive vs. negative immunohistochemical expression of AR). The samples were divided into two groups: positive AR expression (group 1, violet) or negative AR expression (group 0, green), based on the H-score being higher than or equal to 0. The asterisk indicates Kruskal–Wallis < 0.05. The comparison of the methylation in AR CGG of female patients was inconclusive.

Suppl. Figure S2 A) H-score comparison: *MAGEA1*.

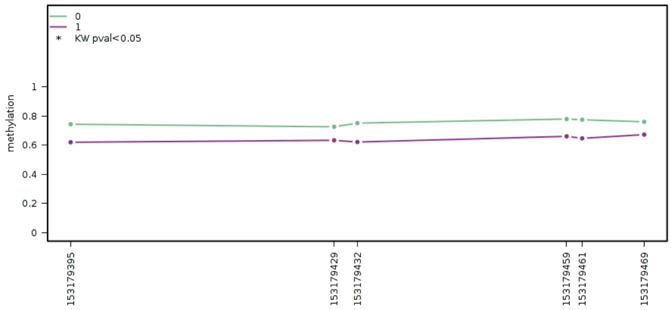

MAGEA1 H-score Males

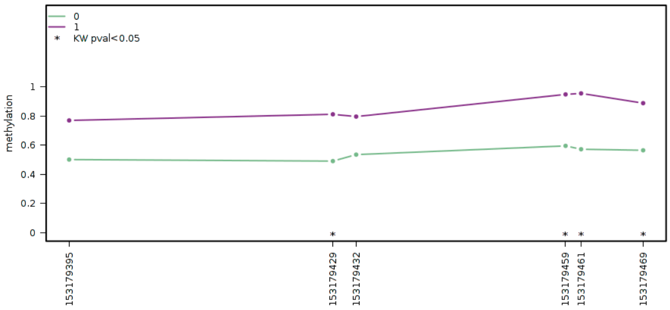

MAGEA1 H-score Females

Suppl. Figure S2. B) H-score comparison: *MAGEA11.*

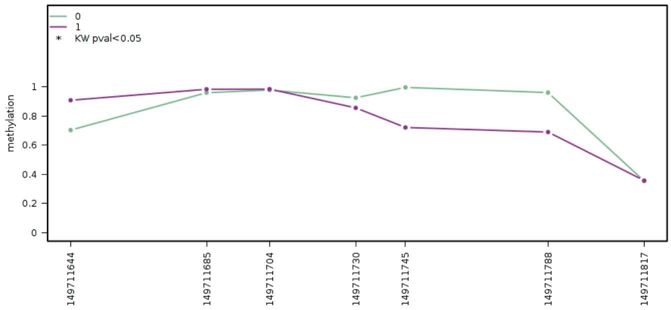

MAGEA11 H-score Males

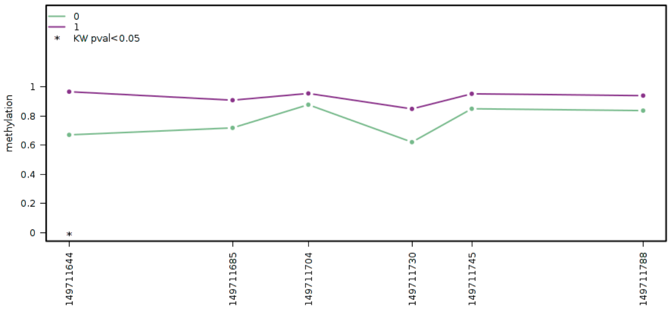

MAGEA11 H-score Females

Suppl. Figure S2. C) H-score comparison: *MAGEC1*.

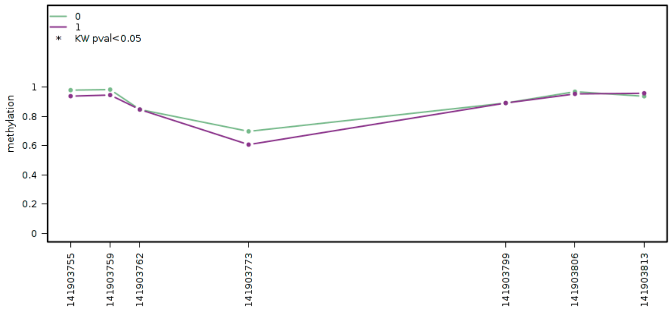

MAGEC1 H-score Males

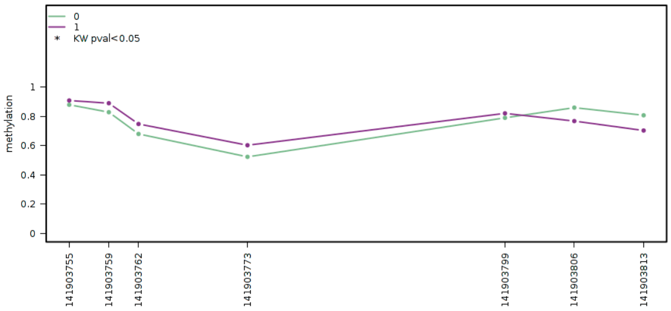

MAGEC1 H-score Females

Suppl. Figure S2. D) H-score comparison: *MAGEC2*.

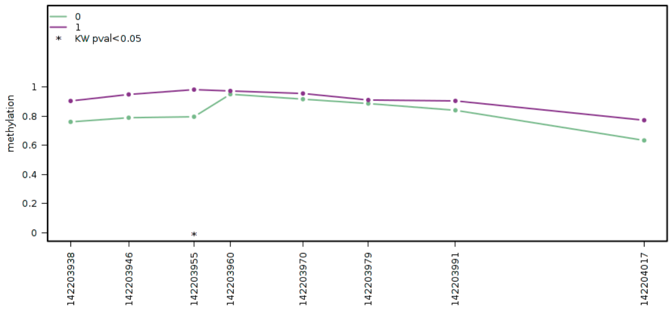

MAGEC2 H-score Males

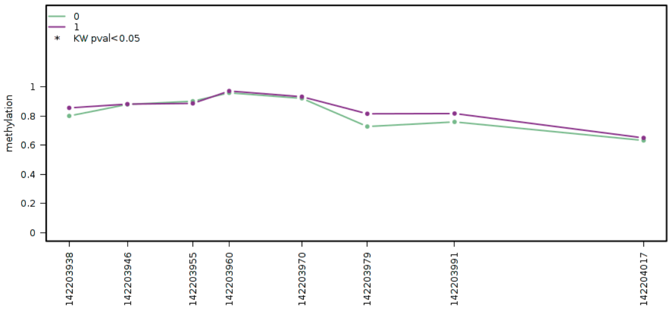

MAGEC2 H-score Females

Suppl. Figure S2. E) H-score comparison: *FLNA*.

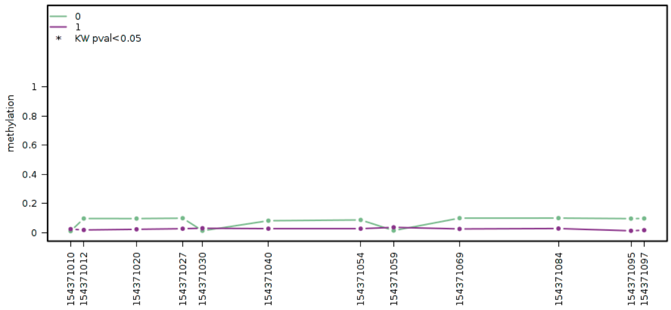

FLNA H-score Males

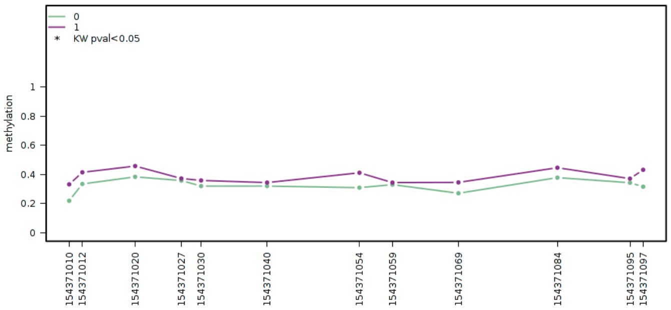

FLNA H-score Females

Suppl. Figure S2. F) H-score comparison: *UXT.*

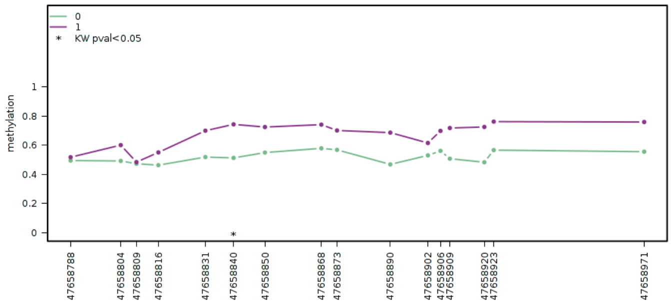

UXT H-score Males

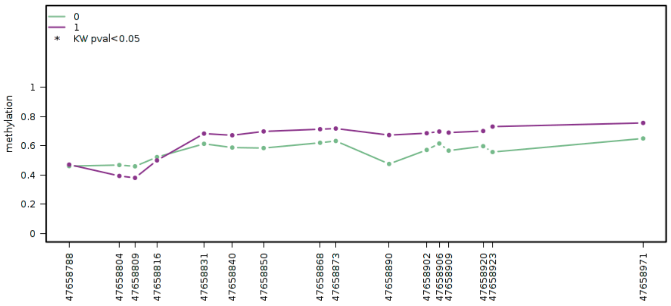

UXT H-score Females

Suppl. Figure S2. A,B,C,D,E,F. DNA methylation analysis genetic of AR regulators on the X chromosome (*FLNA, MAGEA1, MAGEA11, MAGEC1, MAGEC2, UXT*). The samples were divided into two groups: positive AR expression (group 1, violet) or negative AR expression (group 0, green), based on H-score being higher than or equal to 0. The samples were also divided into males and females, given the difference in number of X chromosomes. The asterisk indicates Kruskal–Wallis < 0.05.

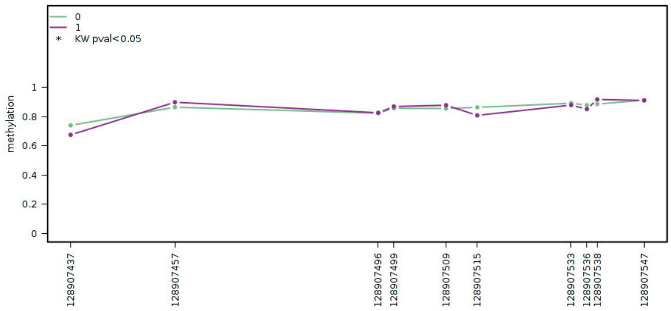

MGMT ENHANCER H-score

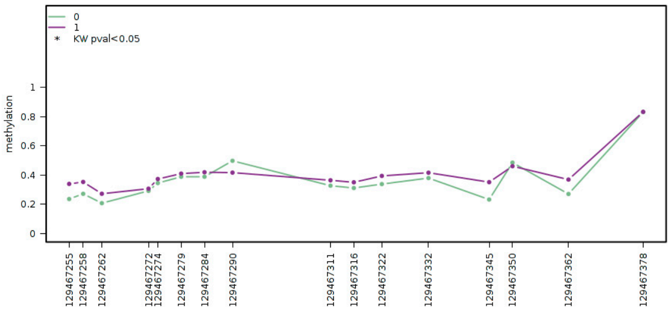

MGMT EXON1 H-score

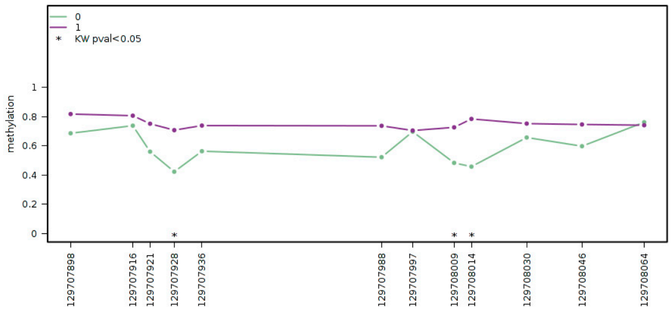

MGMT EX 3 H-score

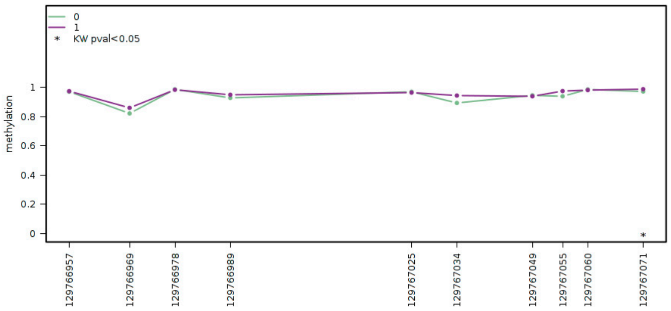

MGMT EX 5 H-score

Suppl. Figure S3. Differential methylation analysis of *MGMT* according to AR expression. The samples were divided into two groups: positive AR expression (group 1, violet) or negative AR expression (group 0, green), based on H-score being higher than or equal to 0. The asterisk indicates Kruskal–Wallis < 0.05. *MGMT* in the region EX 3 is hypermethylated in AR-positive cases (KR < 0.05).

Suppl. Figure S4. A) Grade comparison: *AR* CAG REPEATS.

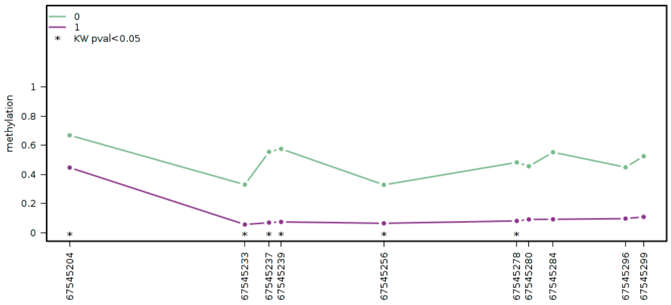

AR CAG REPEATS Grading Males

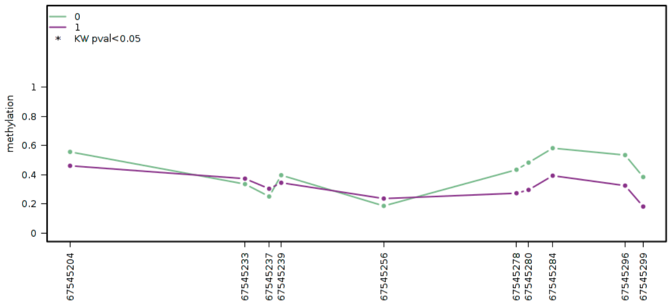

AR CAG REPEATS Grading Females

Suppl. Figure S4. B) Grading comparison: *AR* INTRA CAG-CGG REPEATS.

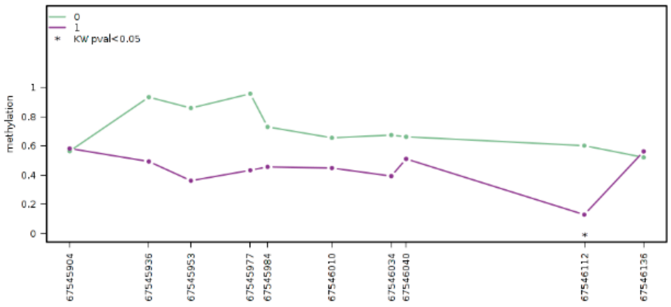

AR INTRA CAG-CGG REPEATS Grading Males

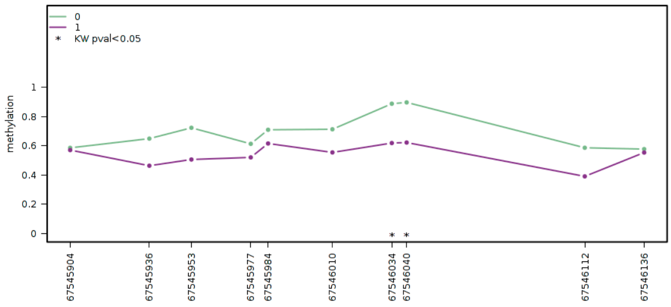

AR INTRA CAG-CGG REPEATS Grading Females

Suppl. Figure S4. C) Grading comparison: *AR* BEFORE CGG REPEATS.

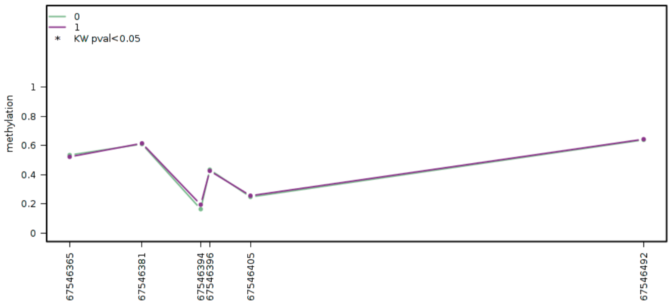

AR BEFORE CGG REPEATS Grading Males

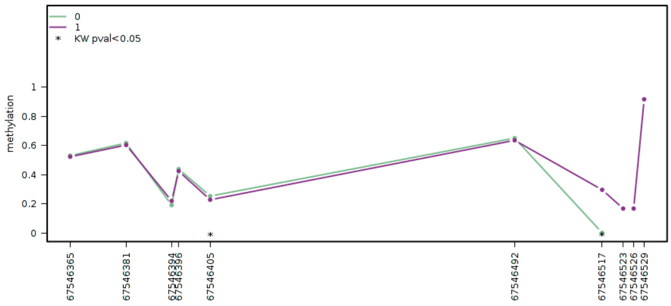

AR BEFORE CGG REPEATS Grading Females

Suppl. Figure S4. D) Grading comparison: *AR* CGG REPEATS.

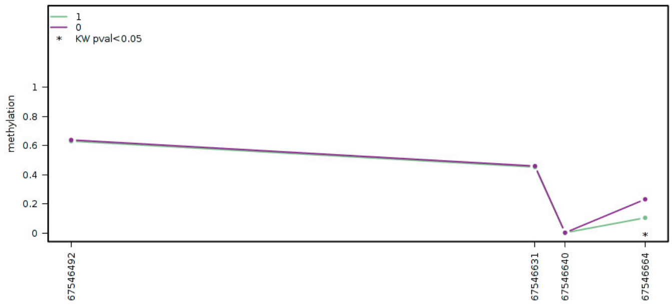

AR CGG Grading Males

Suppl. Figure S4. A, 4B, 4C, 4D). Differential methylation analysis of the *AR* gene promoter region according to pathologic grade. The samples were divided into two groups based on the glioma grade. G2 gliomas were considered low-grade (group 0, green) and G3-G4 gliomas were considered high-grade (group 1, violet). The asterisk indicates Kruskal–Wallis < 0.05. The comparison of the methylation in AR CGG of female patients was inconclusive.

Suppl. Figure S5. A) Grading comparison: *MAGEA1.*

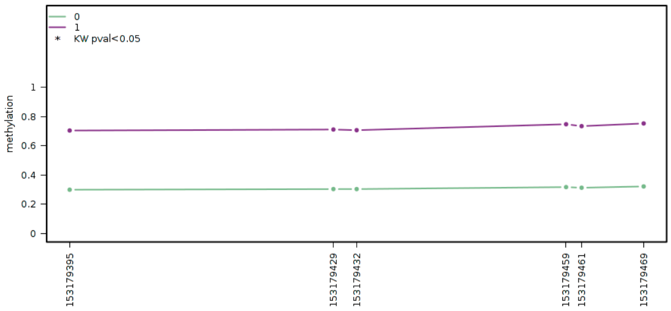

MAGEA1 Grading Males

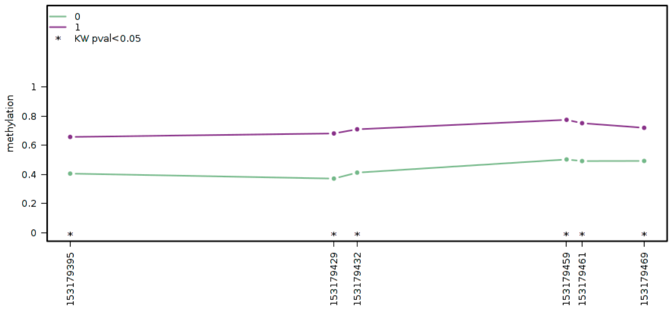

MAGEA1 Grading Females

Suppl. Figure S5. B) Grading comparison: *MAGEA11.*

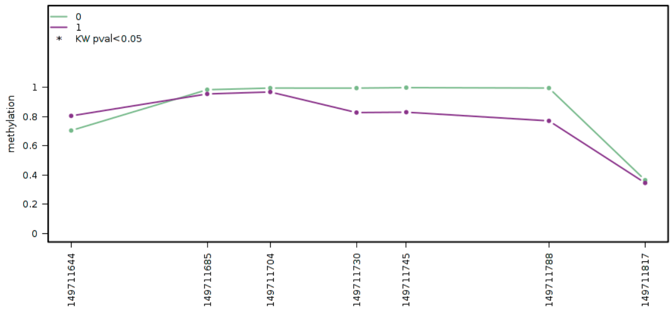

MAGEA11 Grading Males

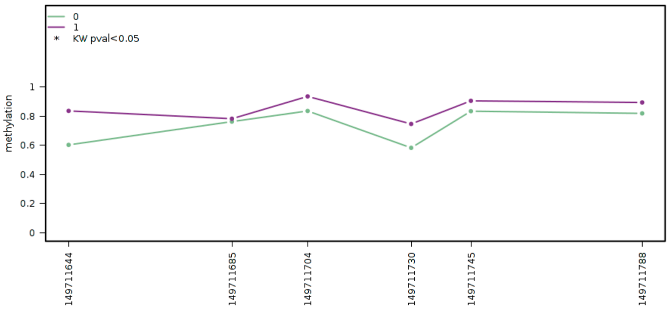

MAGEA11 Grading Females

Suppl. Figure S5. C) Grading comparison: *MAGEC1*.

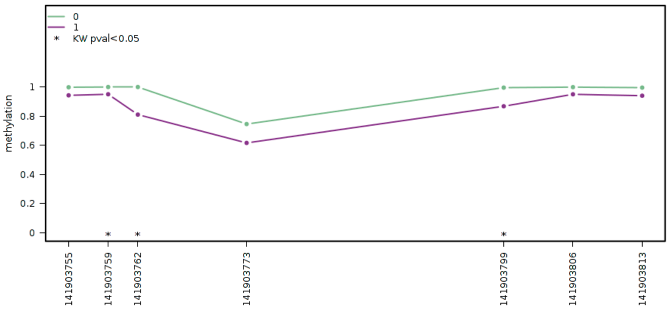

MAGEC1 Grading Males

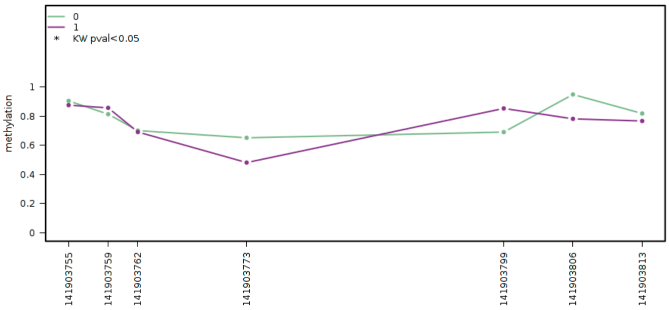

MAGEC1 Grading Females

Suppl. Figure S5. D) Grading comparison: *MAGEC2*.

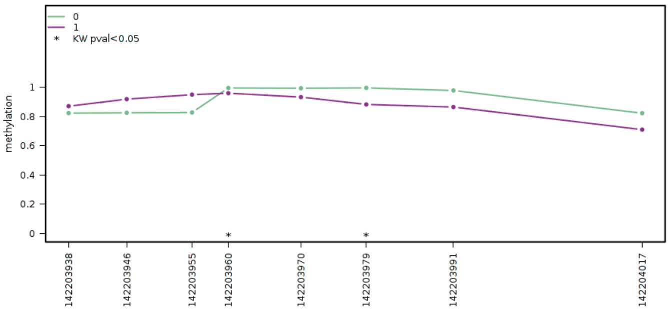

MAGEC2 Grading Males

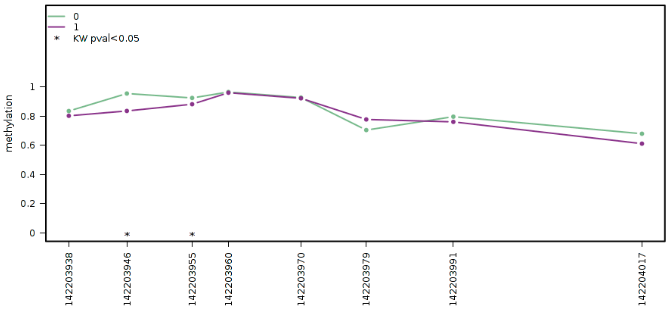

MAGEC2 Grading Females

Suppl. Figure S5. E) Grading comparison: *FLNA*.

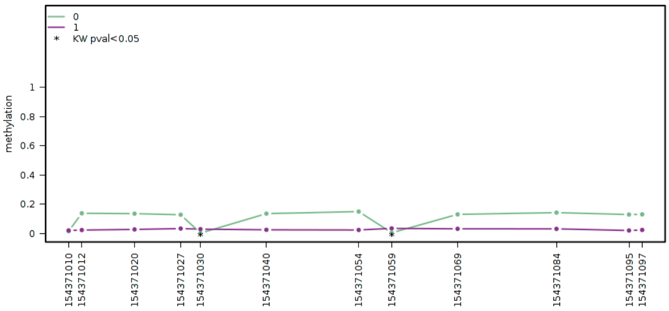

FLNA Grading Males

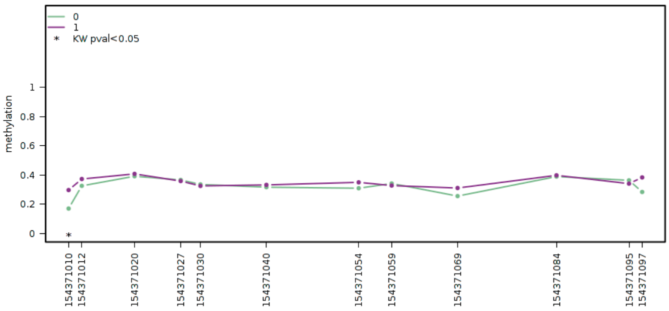

FLNA Grading Females

Suppl. Figure S5. F) Grading comparison: *UXT*.

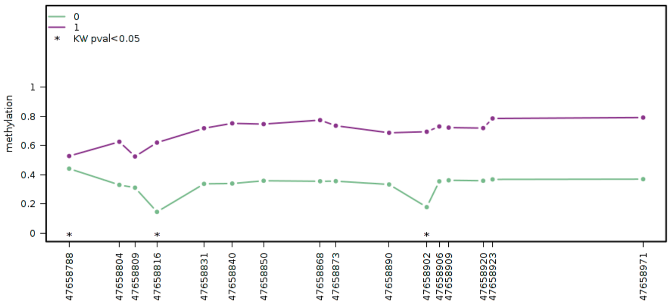

UXT Grading Males

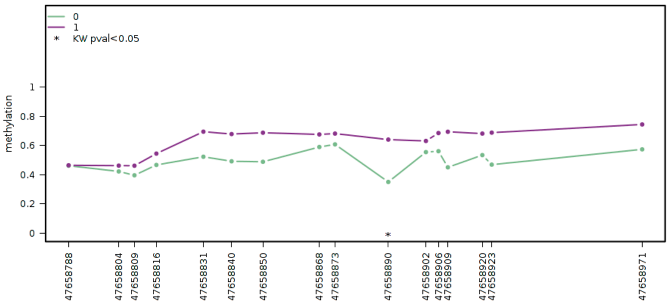

UXT Grading Females

Suppl. Figure S5. A, B, C, D, E, F. Differential methylation analysis of *MAGEA1*, *MAGEA11*, *MAGEC1*, *MAGEC2*, *UXT*, and *FNLA* according to pathologic grade. The samples were divided into two groups based on the glioma grade. G2 gliomas were considered low-grade (group 0, green) and G3-G4 gliomas were considered high-grade (group 1, violet). Since these genes are located on the X chromosome, the samples were also divided into males and females, given the difference in the number of X chromosomes. The asterisk indicates Kruskal–Wallis < 0.05.

Suppl. Figure S6. A) Grading comparison: *MGMT*.

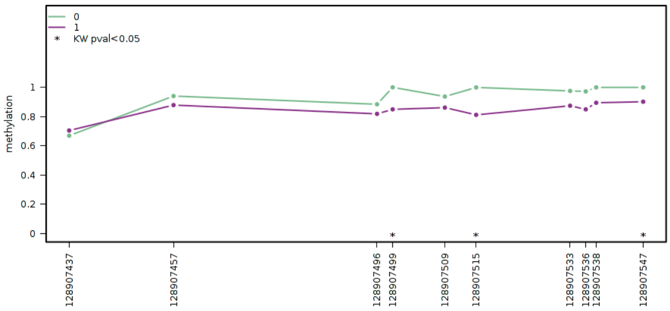

MGMT ENHANCER Grading

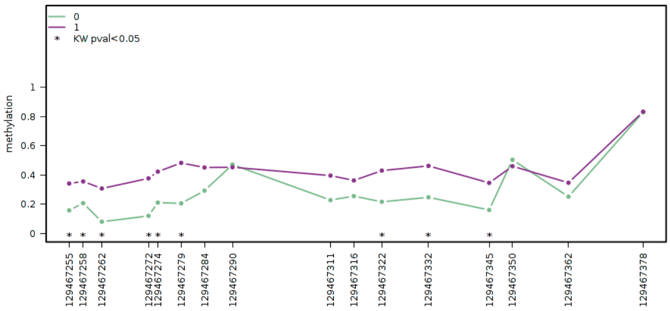

MGMT exon1 Grading

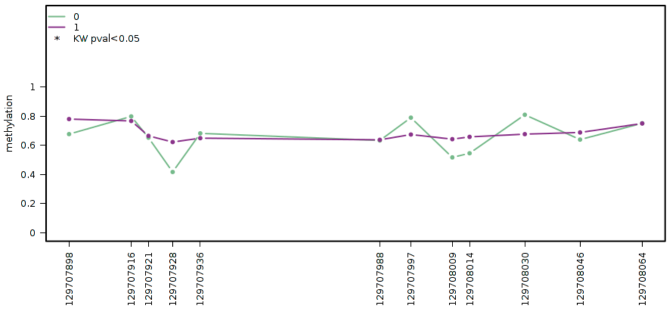

MGMT EX 3 Grading

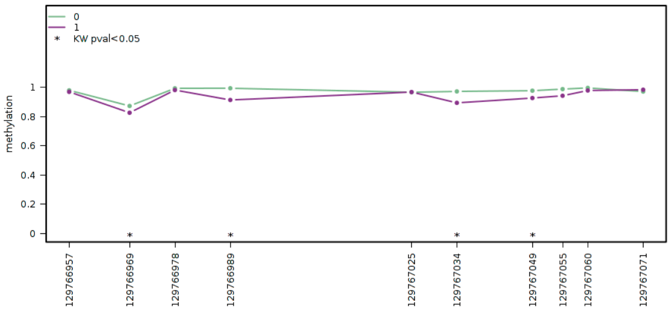

MGMT EX 5 Grading

Suppl. Figure S6. B) Grading comparison: *TERT*.

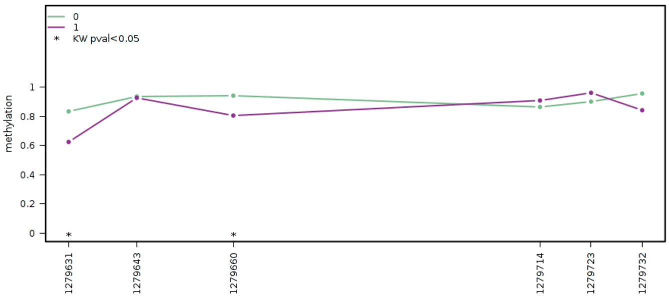

Suppl. Figure S6. A, B. Differential methylation analysis of *MGMT* and *TERT* according to pathologic grade.

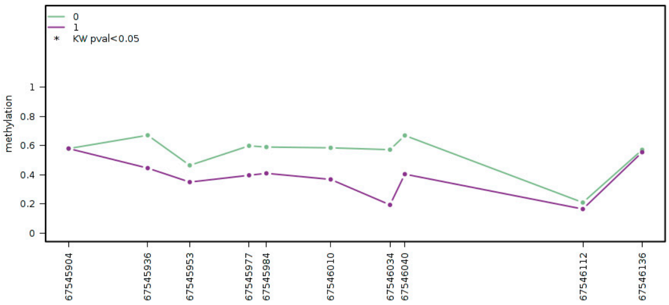

AR INTRA CAG-CGG REPEATS Polyploidy Males

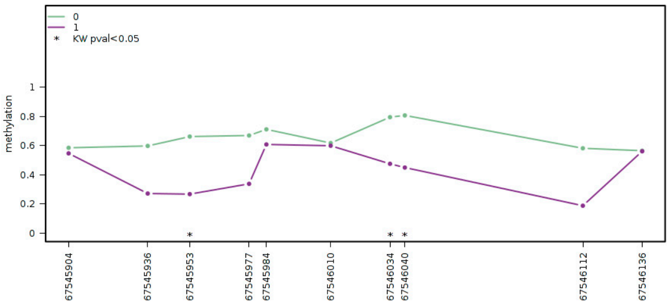

AR INTRA CAG-CGG REPEATS Polyploidy Females

Suppl Figure S7. Polyploidy comparison: *AR* INTRA CAG-CGG REPEATS. Differential methylation analysis of *AR* promoter regions according to X-chromosome copy number. The samples were divided into two groups based on the level of X-chromosome polyploidy. Males were considered "high polyploidy" (group 1, violet) if they had a polyploidy percentage >10%, while they were considered "low polyploidy" (group 0, green) for a percentage <10%.

Suppl. Figure S8. A) Polyploidy comparison: *MAGEA1*.

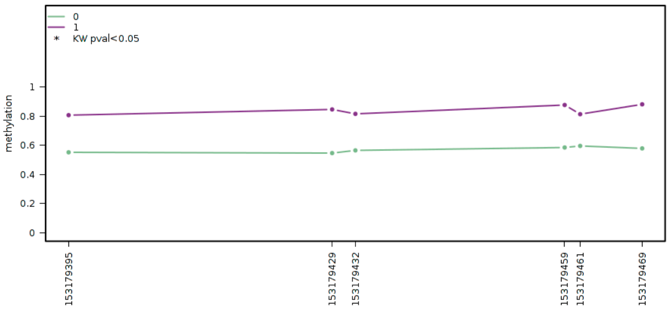

MAGEA1 Polyploidy Males

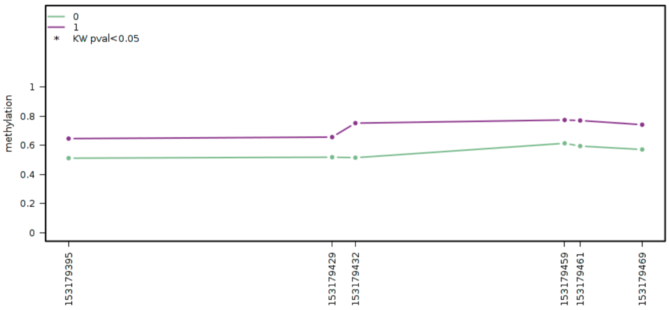

MAGEA1 Polyploidy Females

Suppl. Figure S8. B) Polyploidy comparison: *MAGEA11.*

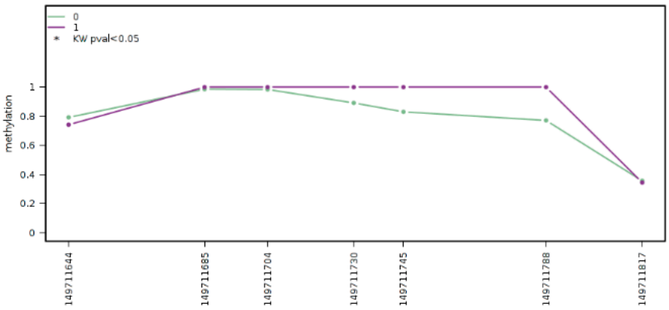

MAGEA11 Polyploidy Males

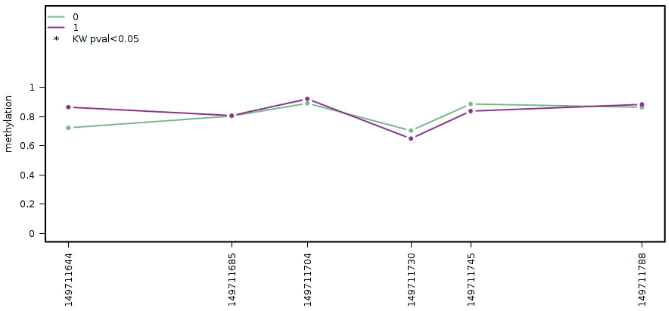

MAGEA11 Polyploidy Females

Suppl. Figure S8. C) Polyploidy comparison: *MAGEC1.*

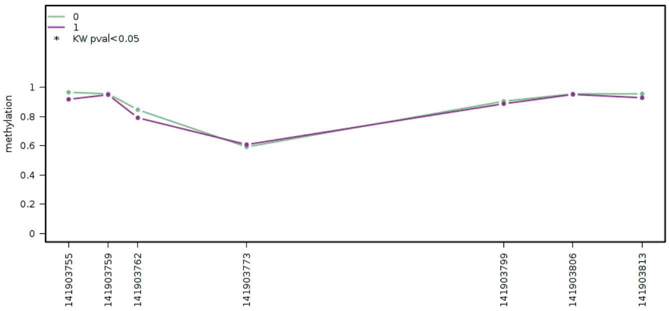

MAGEC1 Poliploidy Males

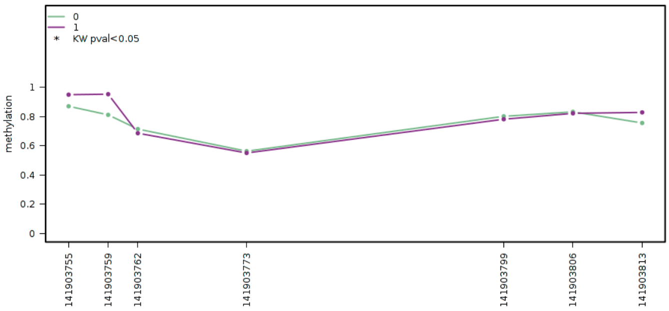

MAGEC1 Polyploidy Females

Suppl. Figure S8. D) Polyploidy comparison: *MAGEC2.*

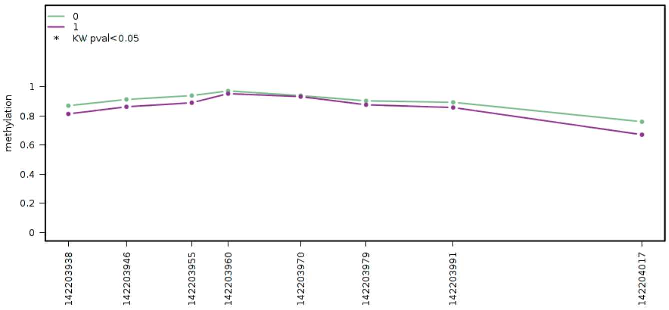

MAGEC2 Polyploidy Males

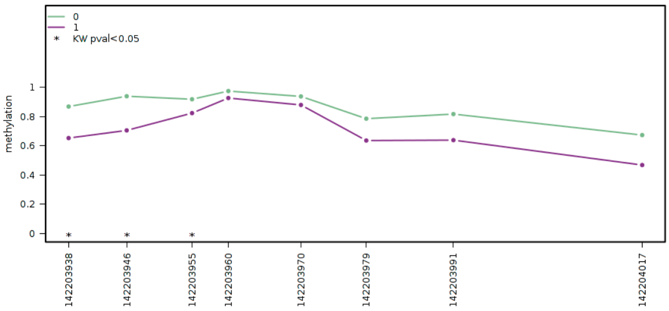

MAGEC2 Polyploidy Females

Suppl. Figure S8. E) Polyploidy comparison: *FLNA.*

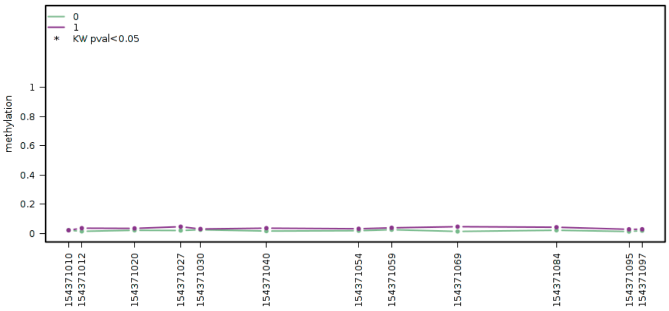

FLNA Polyploidy Males

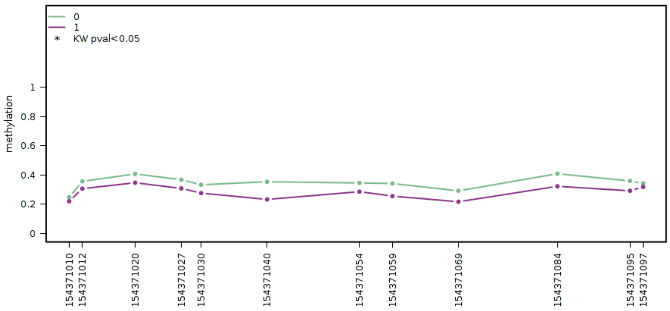

FLNA Polyploidy Felmales

Suppl. Figure S8. F) Polyploidy comparison: *UXT.*

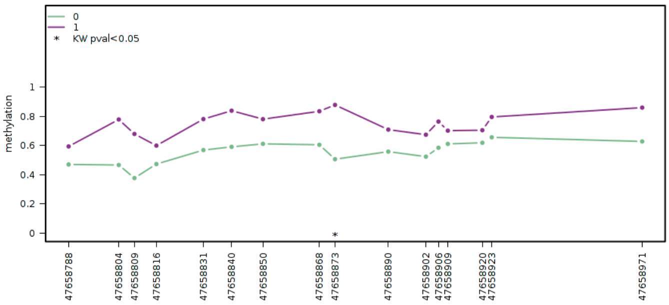

UXT Polyploidy Males

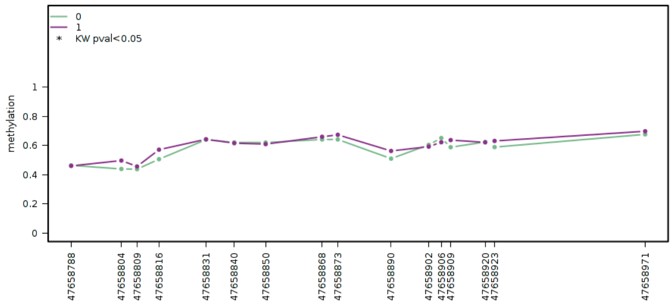

UXT Polyploidy Females

Suppl. Figure S8. A, 8B, 8C, 8D, 8E, 8F. Differential methylation analysis of *MAGEA1, MAGEA11, MAGEC1, MAGEC2, UXT*, and *FNLA* genes according to X-chromosome copy number. The samples were divided into two groups based on the level of X-chromosome polyploidy. Males were considered "high polyploidy" (group 1, violet) if they had a polyploidy percentage >10%, while they were considered "low polyploidy" (group 0, green) for a percentage <10%. Females, on the other hand, had a lower overall level of polyploidy, therefore they were considered "high polyploidy" (group 1, violet) if they had a polyploidy percentage > 0%, while they were considered "low polyploidy" (group 0, green) for a percentage= 0%.

Disclaimer/Publisher's Note: The statements, opinions and data contained in all publications are solely those of the individual author(s) and contributor(s) and not of MDPI and/or the editor(s). MDPI and/or the editor(s) disclaim responsibility for any injury to people or property resulting from any ideas, methods, instructions or products referred to in the content.
